# A rare case of Richter transformation with breast involvement: A case report and literature review

**DOI:** 10.1515/biol-2022-0889

**Published:** 2024-06-18

**Authors:** Wenhui Wang, Hao Chen, Wendong Ju, Weihong Yang, Gaoming Ding, Li Wang

**Affiliations:** Department of Pathology, Hangzhou Women’s Hospital, Hangzhou, Zhejiang, China; Department of Oncology and Hematology, Zhongshan Boai Hospital Affiliated to Southern Medical University, Zhongshan 528403, Guangdong, China; Department of Pathology, Zhongshan Boai Hospital Affiliated to Southern Medical University, Zhongshan, Guangdong, China; Department of Oncology and Hematology, Zhongshan Boai Hospital Affiliated to Southern Medical University, 6 Chenggui Road, East District, Zhongshan, 528403, Guangdong, China

**Keywords:** Richter transformation, breast tumor, chronic lymphocytic leukemia, zanubrutinib, small lymphocytic lymphoma

## Abstract

Richter transformation (RT) represents the development of intrusive lymphoma in individuals previously or concurrently diagnosed with chronic lymphocytic leukemia (CLL) and is characterized by lymph node enlargement. However, cases involving extra-nodal organ involvement as the first symptom are rare. There are no reports of RT with breast lesions as the first symptom. Nonspecific and atypical clinical manifestations represent key challenges in the accurate diagnosis and appropriate treatment of RT. This case report describes an elderly female patient who presented with breast lesions as the first RT symptom. The patient was admitted with a painless mass in the left breast. Examination revealed multiple lymphadenopathies and abnormally high white blood cell levels. The patient was diagnosed with CLL after hematological tests, assessments of bone marrow morphology, and tissue biopsy. Mammography and B-ultrasonography showed solid space-occupying lesions (BI-RADS category 5) in the left breast. Initially, the patient declined a breast biopsy and was therefore prescribed ibrupotinib treatment, which showed limited efficacy. A needle biopsy of the affected breast indicated the presence of diffuse large B-cell lymphoma. Based on auxiliary and pathological examinations and medical history, the final diagnosis was RT with breast involvement. Zanubrutinib with rituximab, cyclophosphamide, doxorubicin, vincristine, and prednisone treatment provided initial control; however, the treatment strategy required adjustment because of the patient’s fluctuating condition. The current status of the patient is marked as stable, showing an overall achievement of partial alleviation. The patient is in the process of receiving follow-up treatment. We also performed a comprehensive literature review on RT, with particular emphasis on its biological paradigm, prognosis implications, existing therapeutic approaches, and emerging directions in treatment modalities.

## 1 Introduction

Richter transformation (RT), previously known as Richter syndrome, is an intrusive transformation, occasionally occurring in chronic lymphocytic leukemia (CLL)/small lymphocytic lymphoma (SLL). About 2–10% of CLL/SLL cases eventually progress to aggressive lymphoma, typically to diffuse large B-cell lymphoma (DLBCL) and occasionally to Hodgkin’s lymphoma [1]. The RT prevalence rate is 0.5–1% per year, often occurring in weak elderly patients and associated with multiple complications and poor prognoses [2]. RT is principally associated with the lymph nodes or bone marrow. Extranodal RT (41%) typically involves organs such as the lungs, gastrointestinal tract, bones, and nervous system [3]. Skin involvement is rare, and breast manifestations have not been reported to date. The pathogenic mechanism associated with RT in CLL is unknown, although immunogenetic studies have suggested the involvement of antigen stimulation [4].

## 2 Case description

**Figure 1:**
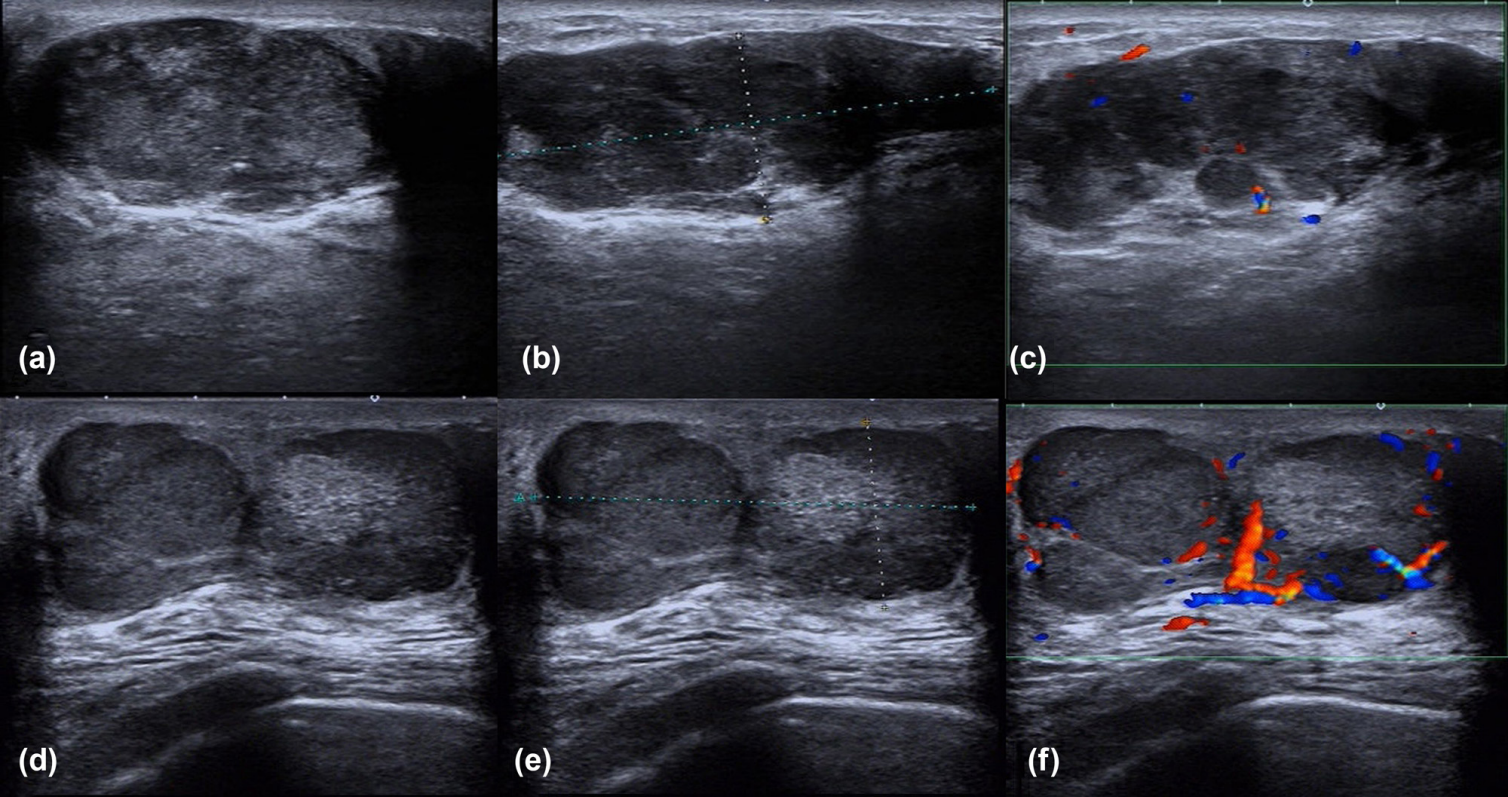
B-ultrasound images before treatment: This shows irregular hypoechoic clusters behind the left papillae, with irregular edges and uneven internal echoes, with parts of the area crisscrossing the surrounding tissue in a canine interlacing pattern, and no changes in echoes seen behind the multiple internal calcification points (a and b). CDFI: low echo mass and short rod-shaped blood flow signal within it (c). Following treatment, a reduction in the irregular hypoechoic mass was observed, with lobular structure and clear boundaries with surrounding tissue (d and e), CDFI: abundant blood flow signals in the hypoechoic mass (f).

**Figure 2:**
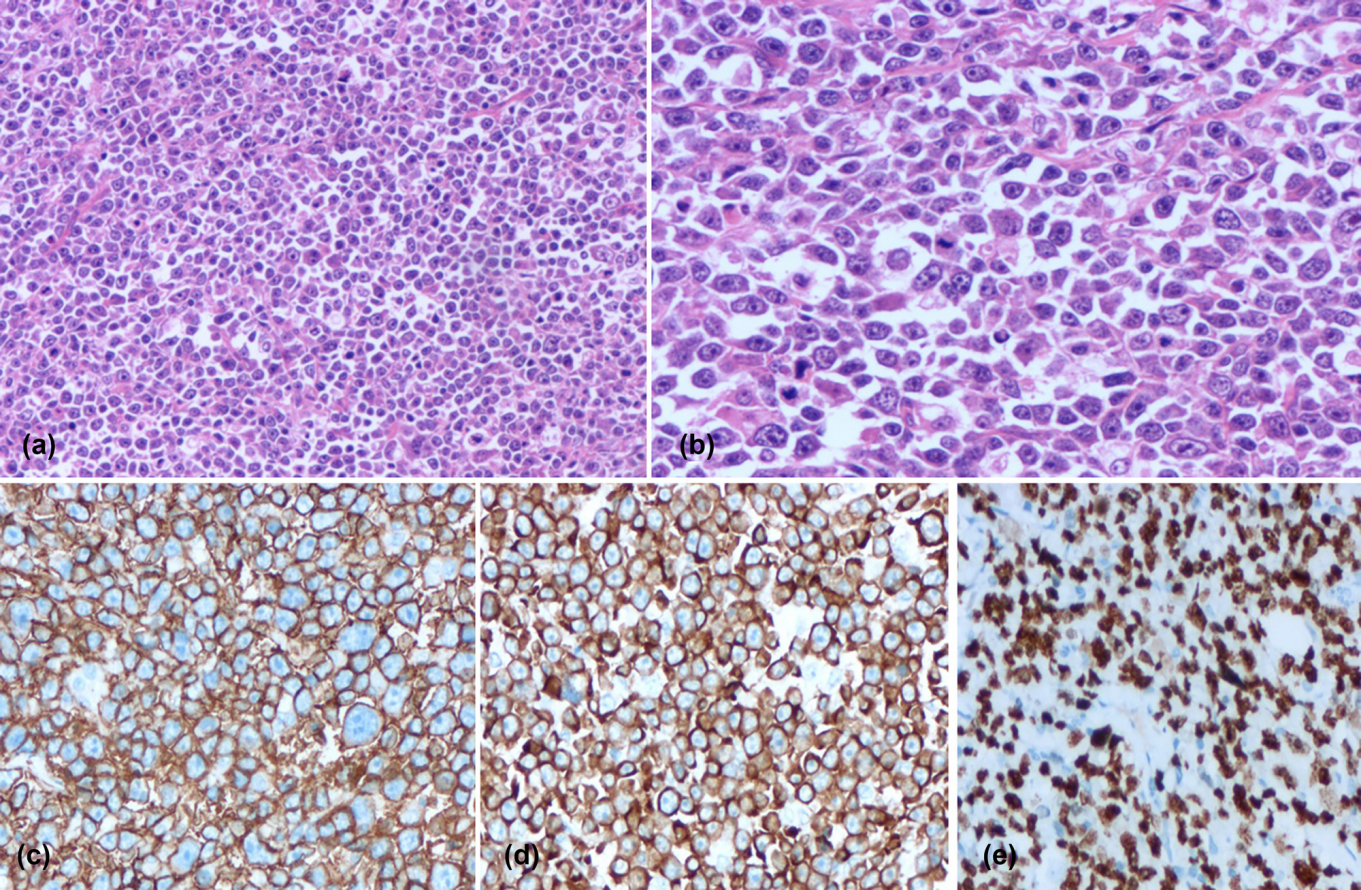
Immunohistochemistry of the needle biopsy from the breast. Hematoxylin and eosin staining demonstrating (a) diffuse and patchy appearance of the tumor cells, with medium to large cell sizes (original magnification ×200); (b) tumor cells showing irregular chromatin, significant nucleoli, and normal mitosis (original magnification ×400). The staining of specific cell markers displayed diffused positive staining for (c) CD20; (d) Bcl-2; and (e) Ki67.

A 76-year-old female patient presented with a complaint of a left breast mass for the past 3 months. No fever, emaciation, night sweating, or other symptoms were reported. In terms of physical examination, full breast examination showed asymmetrical breasts with two palpable masses behind the areola of the left nipple at the 11 o’clock position. The left nipple was slightly dented. The masses had maximum diameters of 8.0 cm, a hard texture, unclear edges, and coarse surfaces; however, no tenderness was identified. The right breast felt pliable without the detection of a definite lump. Blood examinations at admission showed a white blood cell (WBC) count of 89.22 × 10^9^/L; platelets (PLT), 131 × 10^9^/L; hemoglobin (Hb), 105 g/L; neutrophil proportion, 5.4%; lymphocyte proportion, 94.0%; blood lactate dehydrogenase (LDH), 330 U/L; serum β-2 microglobulin, 2,250 Ug/L. The patient was transferred to the Department of Hematology and Oncology after consultation. Further tests revealed mature lymphocyte levels of 95 and 70% in the peripheral blood and bone marrow, respectively. Bone marrow flow cytometry revealed that 69.2% of these were mature B lymphocytes with immunophenotypes of CD19+, CD5+, CD20+dim, CD23+, CD200 partial+, CD79b (−), and FMC7 (−), whereas the levels of immunoglobulin kappa/lambda light chains in the cell membrane were not significant, suggesting the presence of CLL/SLL immunophenotypes (RMH-CLL score: 5 points). Bone marrow biopsy indicated the proliferative activity of nucleated cells (VOL%) as 60%, with significant lymphocyte proliferation. These cells showed a flaky, diffuse distribution and were composed mainly of small mature lymphocytes. Collagen fibrosis was observed in the bone marrow interstitium without osteosclerosis. Immunohistochemistry of the tumor-associated cells showed CD5 (+), CD20 schistose dim (+), PAX-5 (+), CD23 (+), LEF-1 (+), CD200 partial (+), Ki-67 (5%), CD3 (−), CD10 (−), Cyclin-D1 (−), SOX-11 (−), CD138 (−), Kappa (−), and Lambda (−), consistent with CLL (B-CLL; tumor cells accounted for about 70%; MF-1 grade). Monoclonal rearrangement of the IgH gene was positive, and tests for SF3B1 and TP53 gene mutations were negative. Mammography showed that both breasts were multiglandular, with varying densities. Two lumps could be seen in the left breast, with sizes 6.1 cm × 3.9 cm and 2.4 cm × 1.4 cm, respectively, with high density and blurred boundaries. There were several punctate calcifications. Thickening of the skin was observed around the left areola, with retraction and indentation of the left nipple. Patchy asymmetrical density was visible behind the right areola, with a large amount of slightly dense calcification and clustered calcifications. The diagnosis was multiple space-occupying lesions in the left breast (BI-RADS 5) and calcification foci in the right breast (BI-RADS 4A). B-ultrasonography of both mammary glands showed the presence of irregular hypoechoic clusters of about 6.3 cm × 5.3 cm and 2.2 cm × 1.2 cm in size behind the left papillae and beside the papillae at 11 o’clock, respectively (Figure 1a–c). The echo in the right breast was slightly uneven, with scattering in strong echo calcification points. Multiple solid nodules were apparent in the left breast (BI-RADS 5) together with calcification foci in the right breast (BI-RADS 4A). Neck and chest computed tomography (CT) showed multiple enlarged lymph nodes with enhancement on the sides of the neck (the left supraclavicular fossa was significantly enlarged). Bilateral axillary lymph node enlargement was observed, indicating possible lymph node metastasis. Multiple retroperitoneal lymph nodes were also enlarged and enhanced, suggesting a malignant lesion. The bronchus had no significant lesions. The co-existence of CLL and breast cancer was considered in light of the patient’s clinical history and examination results. However, the proposition of a needle biopsy of the breast was declined by the patient. The patient was diagnosed with CLL based on the blood and bone marrow examinations and was treated with ibupotinib (420 mg), a once-daily biologically targeted anti-tumor treatment, as well as with symptomatic treatments, including hydroxyurea to reduce the WBC count. After 2 months of oral administration of ibupotinib, the breast lesions on the left side had not reduced in size but had hardened instead. Furthermore, ecchymosis was found on the nodule without tenderness. A needle biopsy was subsequently performed on the left breast after further communication with the patient and her family. Pathological analysis showed an absence of normal gland ducts in the breast. The tumor infiltrate showed a diffuse growth pattern consisting of large cells with mostly immunoblastic appearance (large nuclei with coarse chromatin, prominent nucleoli, and basophilic or vacuolar cytoplasm), together with increased mitotic activity (Figure 2a and b). The levels of immunohistochemical markers on the tumor cells showed LCA (+), CD20 (+), CD79a (+), Bcl-2 (+), Bcl-6 (30%+), MUM1 (+), FLI-1 (+), CD15 (focal+), Ki-67 (hotspot 90%+), P53 overexpression (+), CD5 (−), CD10 (−), CD23 (−), Cyclin D1 (−), PD-1 partial (+), CD21 (−), CD30 (−), and Epstein-Barr virus (EBV) (−). Tests for MYC, Bcl-2, and Bcl-6 gene rearrangements (fluorescence in situ hybridization) were negative (Figure 2c–e). The breast lesions were confirmed to result from DLBCL with a nongerminal center B-cell-like population. The patient received two cycles of chemotherapy with a regimen consisting of the Bruton’s tyrosine kinase (BTK) inhibitor zanubrutinib, combined with rituximab, cyclophosphamide, doxorubicin, vincristine, and prednisone (R-CHOP) based on the medical history and auxiliary and pathological examinations.

**Figure 3:**
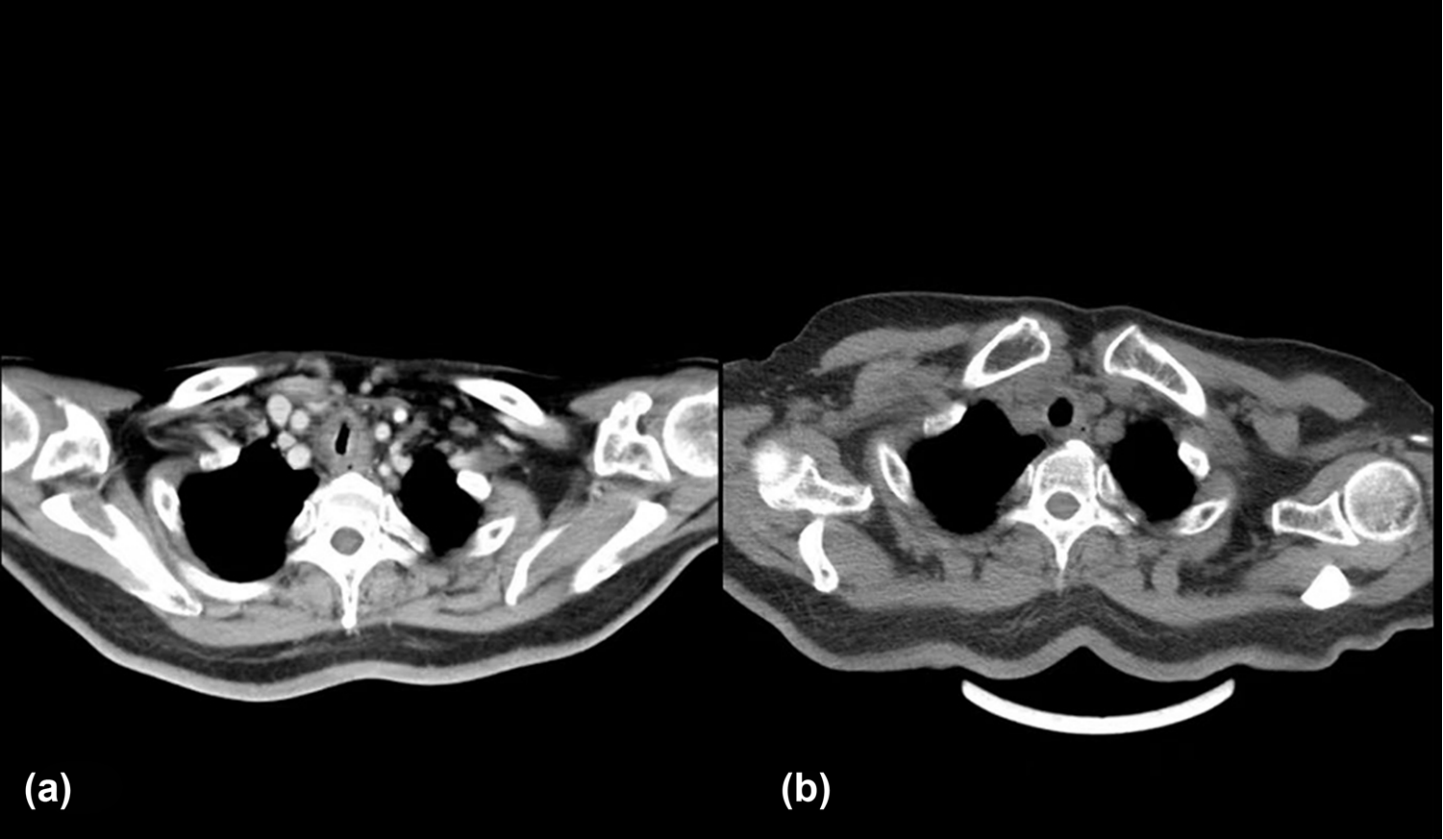
CT scan of tracheostenosis before (a) and after (b) changing the treatment regime for RS with breast involvement and tracheal invasion. (a) Manifestations of severe tracheostenosis. (b) Significant improvement in tracheostenosis.

**Figure 4:**
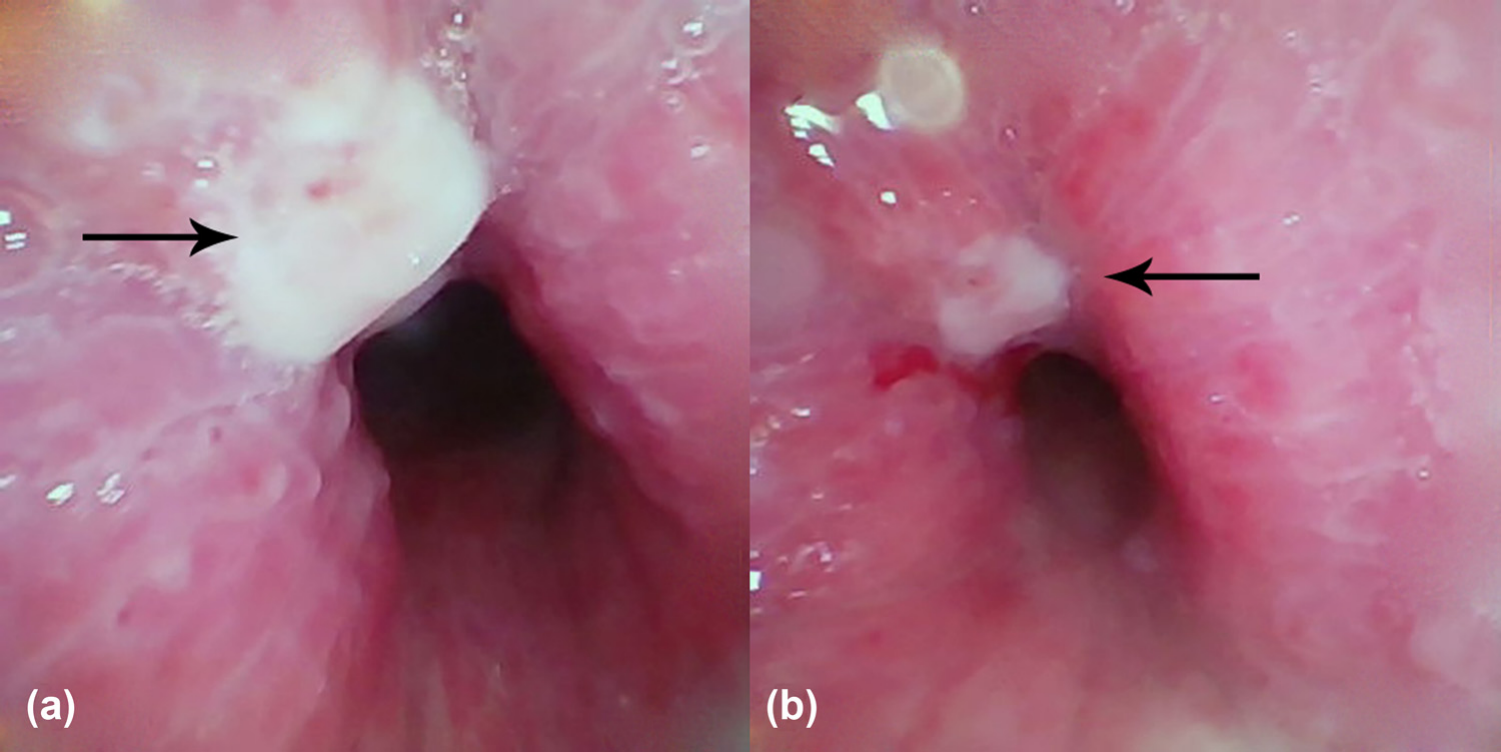
Tracheoscopy of the middle and upper trachea. (a) Local swelling and stenosis can be observed on the tracheal wall, with a 1-mm × 1-mm mass at the upper right portion protruding from the tube wall (black arrow). (b) Enlargement of the view of the white necrotic areas on the tracheal surface.


After six cycles of treatment, the sizes of the tumors in the left breast were reduced from 6.3 cm × 5.3 cm (Figure 1a–c) to 4.5 cm × 3.3 cm (Figure 1d–f) and from 2.2 cm × 1.2 cm to 1.2 cm × 0.9 cm, respectively. The sizes of the involved lymph nodes were significantly reduced, and the patient showed partial response. On re-examination, the blood parameters were 4.0 × 10^9^/L WBC, 88.8% neutrophils, 107 g/L Hb, 164 × 10^9^/L PLT, 231 U/L LDH, and 1,463 Ug/L serum β-2 microglobulin, indicating significant improvement. The bone marrow morphology was completely normal. The patient achieved stable disease after three cycles of chemotherapy with zanubrutinib and R-CHOP with no further improvements in her condition. During chemotherapy, the patient developed cough, sputum, and asthma due to Grade IV myelosuppression, resulting in irregular administration of chemotherapy. The cause of these symptoms was evaluated by performing neck and chest CT, which showed focal stenosis at the trachea-thoracic entrance. This narrowed the local airway to 7.4 mm × 5.2 mm (lung window measurement) and thickened the surrounding soft tissues, suggesting tumor invasion (Figure 3a). Tracheoscopy showed focal stenosis and a suspicious mass in the middle and upper trachea, suggestive of tumor infiltration (Figure 4). Biopsy was not performed to avoid bleeding, edema, and other life-threatening complications. The patient developed significant shortness of breath and was given one course of rituximab, etoposide, prednisone, vincristine, cyclophosphamide, and doxorubicin (R-EPOCH) chemotherapy with decreased dosage. CT re-examination 2 days after the R-EPOCH treatment showed that the focal stenosis at the thoracic entrance of the trachea had improved significantly, and the narrowest part of the trachea had increased in size to 11 mm × 7 mm from the previous 7.4 mm × 5.2 mm (lung window measurement) (Figure 3b). The patient’s cough and shortness of breath were significantly relieved. After continuous R-EPOCH treatment, the patient’s condition improved, and the respiratory symptoms disappeared. Currently, the patient is still in follow-up.

**Informed consent:** Informed consent has been obtained from all individuals included in this study.
**Ethical approval:** The research related to human use has been complied with all the relevant national regulations, institutional policies, and in accordance with the tenets of the Helsinki Declaration and has been approved by the ethics board of Hospital of Zhongshan Bo’ai Hospital, approved the protocol of the study on March 8, 2023 (No. KY-2023-03-01).

## 3 Discussion

RT is a rare hematopathology, defined by the World Health Organization (WHO) classification of tumors of hematopoietic and lymphoid tissues as the development of an aggressive lymphoma arising in the background of CLL or SLL [5].

RT is considered when patients with CLL show rapid clinical deterioration, new manifestations such as weight loss and fever without infection, organ dysfunction caused by asymmetric lymph node enlargement with or without intrusive or obstructive tumor growth, and an abrupt excessive increase in LDH [6]. RT may manifest in extranodal sites and thus extranodal masses should be examined as a differential diagnosis in patients with CLL [4]. The clinical manifestations of the patient in the present case study were consistent with the above characteristics. Histopathological examination of biopsy samples is crucial for diagnosing RT. However, a fine-needle biopsy or an aspirated specimen cannot fully represent the pathological structure of the tumor, especially in cases where sheets of transformed cells are mixed with small cells, resulting in false-negative results. Enlarged proliferation centers can occasionally be observed in some cases of progressive or aggressive CLL by fine-needle biopsies of the lymph nodes, which may result in false-positive misdiagnosis of RT [7]. RT usually does not occur in all lymph nodes or extranodal organs but is limited to the transformation of a single locus. Therefore, the selection of an appropriate biopsy site is essential. In patients with clinical features suspicious of RT, the standardized uptake value of F-18 fluorodeoxyglucose (^18^F-DG) positron emission tomography (PET)/CT may guide the selection of the biopsy site as RT-affected sites may overlap with DLBCL development. Any biopsy exploring RT occurrence should focus on lesions with the largest imaging diameters, fastest progression dynamics, and/or most active lesion shown by increased ^18^PET/CT ^18^F-DG uptake [8].

The clonal relationship with CLL should be assessed by comparing rearrangements in immunoglobulin genes during the CLL and RT stages if the biopsy indicates the presence of aggressive lymphoma. The majority of DLBCL-type RT cases (80%) exhibit clonal relatedness with the preceding CLL stage, indicating that the immunoglobulin heavy chain variable region IGHV-D-J gene rearrangements are related before and after transformation, thereby representing true transformation. In contrast, a small subset of RT cases displays immunoglobulin gene rearrangements distinct from the CLL stage, indicating a lack of clonal relatedness and suggesting the development of de novo lymphoma in patients with CLL [9,10]. As 47% of patients with RT transform before CLL treatment [9] and in the absence of CLL stage material or archival data, classification of the clonal relationships may not always be feasible. Alternatively, the fixation of RT biopsy tissue in formalin may result in material that is unsuitable for molecular studies [11]. In the present case, rational interpretation of gene rearrangements was challenging, as both CLL and DLBCL were found with an absence of sequential disease progression, together with a lack of molecular findings in tissue specimens from the CLL stage. The biopsy specimen was fixed with formalin and despite repeated molecular investigations, satisfactory DNA amplification was not successful, possibly because of the small quantity of the biopsy tissue, delayed sealing of the wax block, and the high rate of DNA degradation in the formalin-fixed tissue. Such early transformation events may lead to significant divergence between the primary and transformed tumor and preclude any correlative inference.

Due to different diagnoses and treatment schemes, CLL transformation into RT requires differentiation from progressive and invasive CLL, which may be associated with lymphoproliferative center fusion and agglomerated proliferative cell expansion [5,12]. From a pathological standpoint, DLBCL-type RT is characterized by confluent sheets of large neoplastic B lymphocytes resembling either centroblasts or immunoblasts [4,10]. Aggressive CLL shows increased sizes and proliferative activities of the tumor cells together with expanded proliferation centers in the lymph nodes, which reach sizes exceeding 20 times that of the field, resulting in confluence and increased numbers of proliferating cells [5,12]. Because the current WHO classification does not provide criteria supporting the differentiation between histologically aggressive CLL and DLBCL-type RT, the differential diagnosis is currently based on the individual experience and expertise of the pathologist. The existing proposed criteria for the differentiation of DLBCL-type RT from histologically aggressive CLL are as follows: [13] (1) large B cells with nuclei equal to or larger than macrophage nuclei or more than twice those of normal lymphocytes and (2) a diffuse growth pattern involving large cells rather than diffusion only in a small locus. In terms of immunophenotypes, CD20 is commonly present in DLBCL-RT, with CD5 presence observed in some cases (30%) and CD23 more rarely (15%), consistent with the immunophenotype of the present patient. Most DLBCL-RT (90‒95%) exhibit a post-germinal phenotype (IRF4-positive), whereas only 5‒10% show a germinal center phenotype (CD10 expression) [14]. Notably, CD5 and CD23 expression occurs in cases with a demonstrable lack of clonal relatedness [15,16]. Programmed cell death 1 (PD-1) is only expressed in the proliferative center of para-immunoblasts and is common in DLBCL-RT, whereas it is rarely observed in *de novo* DLBCL [16]. He et al. reported that PD-1 expression in RT-DLBCL might represent another clonal relationship surrogate when considered alongside CD5/CD23 loss [16]. RT of the DLBCL type also commonly expresses Bcl-2. However, except for rare cases with Bcl-2 amplification, pathological mutations are rarely observed in the Bcl-2 gene in these cases, whereas translocations or somatic mutations are frequently observed in de novo DLBCL [11]. Although rarely observed in CLL and de novo DLBCL, increased PD-1 levels commonly occur in up to 80% of DLBCL-RT cases [16]. The majority of DLBCL-RT cases are negative for EBV2 and can be distinguished from primary EBV-positive DLBCL [10].

Genetic characteristics that contribute to the aggressiveness and chemotherapy resistance of RT include mutations or deletions in TP53, NOTCH1, MYC, and/or CDKNA [10,17,18]. TP53, the primary regulator of the DNA damage response pathway, is one of the most commonly mutated genes in DLBCL-RT and is found in about 60% of cases during the transformation phase. The TP53 deletion phenotype can be the prognostic marker of chemotherapy refractoriness in RT [10,18]. The degree of NOTCH1 mutation is a confirmed transformation risk factor, and the probability of RT in individuals with CLL and NOTCH1 mutation (45%) is significantly higher than that in patients with CLL without these mutations [19]. In addition, Kohlhaas et al. reported that Akt activation initiates the transformation of CLL to aggressive lymphoma by inducing NOTCH signaling between RT cells and T cells in the tumor microenvironment [20]. A recent study by Tsagiopoulou et al. on the whole-genome chromatin profiling of histone 3 lysine 27 acetylation in 22 CLL cases from major subsets found that subset 8 displayed a remarkably different profile of chromatin activation. This CLL subset was also notable for having the highest risk of RT among all the CLL subsets. This finding provides additional clues for deciphering the underlying molecular basis of the clinical behavior [21].

Patients with DLBCL-RT have a poor prognosis. The risk factors associated with poor prognosis include a Zubrod performance status score >1, an abnormally increased LDH level, PLT count ≤100 × 10^9^/L, tumor size ≥5 cm, and progression beyond second-line treatment regimens [22]. Nevertheless, the best prognostic index is the clonal association between the transformed DLBCL and the original CLL. Patients with clonal correlations between their RT and CLL tumors typically show shorter median survival (8–16 months) than patients without such correlations (5 years) [10,23]. Therefore, studying clonal relationships between DLBCL-RT tumors in patients with CLL, especially clonally unrelated DLBCL that may be managed as de novo, rather than by CLL transformation, is of clinical importance [24].

In the present case, the patient initially presented with a breast lump, and concurrent CLL and DLBCL were discovered. Owing to the lack of the availability of pre- and post-transformation immunoglobulin gene samples for comparison, clonal typing was not possible. We considered the possibility that CLL occurred first, followed by DLBCL transformation after a certain time. CLL shows morbidity concealment, and 25% of patients show no significant symptoms at the early stage. In the present case, the response to ibupotinib treatment was initially poor, with better outcomes when the patient was switched to R-CHOP combined with zanubrutinib. This combination treatment was initially effective; however, it subsequently became ineffective, showing bone marrow suppression and trachea invasion. Switching to R-EPOCH led to remission albeit with a long plateau, indicative of a poor prognosis and implying an association between the unfavorable prognosis and treatment-resistant nature of RT, with a clonally related subtype involving CLL. Currently, the long-term condition of the patient requires further follow-up.

No universal treatment has been reported for RT, and current regimens include chemotherapy, targeted therapies, and hematopoietic stem cell transplantation (SCT) [25]. Combination chemotherapy containing rituximab is the most frequently used therapy for DLBCL-RT. The R-CHOP regimen has a remission rate of 67% (complete remission, 7%), an average progression-free survival (PFS) of 10 months, and a median overall survival (OS) of 21 months [26]. R-EPOCH was found to show a 37% remission rate (20% of patients relieved) in patients with DLBCL-RT, with a median PFS and OS of 3.5 and 5.9 months, respectively [27]. R-CHOP or R-CHOP-like regimens (i.e., R-EPOCH) have been widely employed as the first option for treating DLBCL-RT because, unlike other regimens, they can achieve a balance between activity and toxicity [11].

Owing to the short remission period of chemotherapy alone, autologous and allogeneic SCT has been proposed as a post-remission treatment option for patients with DLBCL-RT [21]. Multidrug chemotherapy containing rituximab plays a crucial role in RT induction therapy. Patients who respond to induction therapy should receive SCT to prolong survival. Generally, younger patients (<60 years old) receiving low-intensity treatments survive longer after allotransplantation. Recently, several newer treatment modalities have been explored for use in the management of RT. These include traditional chemoimmunotherapy regimens combined with targeted agents such as BTKi and BCL2i; immunotherapy combined with targeted agents; non-covalent BTKis; bispecifc T-cell engagers; and chimeric antigen receptor T-cell therapy. In addition, various novel targeted agents are currently under investigation in phase 1 and 2 clinical trials for the treatment of RT. Although some progress has been made toward the optimization of the treatment of RT, further study is needed to evaluate the long-term outcomes of recently published trials and test the efficacies of novel agents [28–30].

## 4 Conclusions

RT with breast involvement is extremely rare and can easily lead to misdiagnosis or delayed diagnosis. For patients presenting with generalized lymphadenopathy and extranodal manifestations, the expeditious and precise selection of the site of RT transformation for pathological biopsy is critical to ascertain a definitive diagnosis. The evaluation of clonal relatedness is highly significant for both the prognosis and therapeutic management of the patient. Moreover, chemotherapeutics combined with BTK inhibitors may benefit patient survival. SCT shows a positive prognostic significance for younger patients, who are sensitive to induction therapy. Owing to the rarity of RT with breast involvement, randomized investigations using large cohorts are not possible. Thus, more case studies and analyses are necessary to advance the diagnosis and treatment of this rare disorder. Further understanding of the underlying tumorigenic mechanism, molecular detection, and gene-targeted therapy may provide novel directions for treating this rare condition.
